# Determination of the Effects of Prone Position on Oxygenation in Patients with Acute Respiratory Failure Under Mechanical Ventilation in ICU

**DOI:** 10.25122/jml-2018-0028

**Published:** 2018

**Authors:** Simin Jahani, Ziba Hajivand Soleymani, Marziyeh Asadizaker, Farhad Soltani, Bahman Cheraghian

**Affiliations:** 1. Nursing and Midwifery School, Ahvaz Jundishapur University of Medical Sciences, Ahvaz, Iran; 2. Department of Anesthesia and Intensive Care Unit, School of Medicine, Ahvaz Jundishapur University of Medical Sciences, Ahvaz, Iran; 3. Health School, Ahvaz Jundishapur University of Medical Sciences, Ahvaz, Iran

**Keywords:** Prone position, supine position, acute respiratory failure, mechanical ventilation, ARF: Acute Respiratory Failure, ABG: Arterial Blood Gas, Pao_2_: Pressure of Arterial Oxygen, MV: Mechanical Ventilation, ICU: Intensive Care Unit, SIMV: Synchronized Intermittent Mechanical Ventilation, Sao_2_: Saturation of Oxygen Arterial Blood

## Abstract

**Introduction:** Patients under mechanical ventilation are usually in the supine position due to various arterial/venous tubes attached to them. Although many studies emphasize the advantages of prone position for oxygenation, some studies enumerate its disadvantages.

**Objective:** The aim of the present research was to determine the effect of prone position on oxygenation of patients with Acute Respiratory Failure (ARF) under mechanical ventilation in the Intensive Care Unit (ICU).

**Methods:** The present study is a single-group clinical trial (pretest-posttest), which was performed in 2017 in Ahvaz, Iran. The population of the study included 58 patients with ARF under Mechanical Ventilation (MV) in ICU in the Golestan Hospital of Ahvaz City. Patients were asked to lie in a supine position for 2 hours, and their physiological signs were measured twice at one-hour intervals. Arterial Blood Gas was tested at the end of the second interval. Afterward, they lied in the prone position and their physiological signs were again measured twice at one-hour intervals and Arterial Blood Gas (ABG) was tested at the end of the second interval. Collected data were statically analyzed by SPSS v.22.

**Findings:** The prone position had a significant relation to Oxygen Arterial Blood (Sao_2_) and Pressure of Arterial Oxygen (pao_2_), (p-value<0.05). Also, on days one and two, there was no significant relationship between the prone position and physiological signs; however, this relation was significant on day three (p-value<0.05).

**Conclusions**: Results showed that the prone position improves sao_2_ and pao_2_ without adverse effect on physiological signs.

## Introduction

When lungs are unable to exchange enough gas, acute respiratory failure occurs. This illness can rapidly progress to fatality if it is not attended to. The hypoxic respiratory failure refers to respiratory failure along with Hypoxia (type 1); while the hypercapnic respiratory failure is a ventilation disorder (type 2). These are associated with decreases in pao_2_ and paco_2_ respectively.

Acute Respiratory Failure (ARF) is a common cause of hospitalization in the Intensive Care Unit. In some ICUs, more than 75% of patients need Mechanical Ventilation (MV) during the hospitalization period. The ARF causes fatality for 33 to 37% of patients under MV [2]. Fatality factors include old age, aggravation of progressive multiple organ dysfunction, pulmonary and non-pulmonary illnesses, and acidosis [3].

Patients with respiratory failure having symptoms such as acute respiratory distress (more than 30 respirations/min, mental transformations, impaired judgment, delusion, sleepiness and hemodynamic instability) need intubation and MV. In these cases, there is no need to wait for Arterial Blood Gas (ABG) tests and any delay in treatments may be hazardous [1]. Deteriorating gas exchange, non-responsiveness to supportive measures, and respiratory distress are the main causes of using MV in patients with acute respiratory failure [2].

On this issue, Black and Hawkes write: “Although mechanical ventilation is a saving measure and improves gas exchange, but just like other measures, these would not be achieved without a price” [4].

Since various tubes and MV apparatus are attached to patients in ICU, they are static most of the time. So, they mostly lie in a supine position. Also, despite the risk of bedsore, convenience of nursing operations in the supine position may encourage the nursing personnel to keep the patient in the same position for more extended periods [5]. On the other hand, immobility affects all the organs of the body, and it reduces the expansibility of the lungs, weakens the respiratory muscles, and accumulation of secretions. This creates atelectasis and hypostatic pneumonia, specifically in the lower parts of the lung [6].

In order to prevent the consequences of immobility, the position of the patient is required to be changed frequently. Studies have been made on changing patient’s position to improve oxygenation, specifically for those who are under MV and may encounter its side effects. Miller was the first to mention the advantages of lying in a prone position, rather than supine position. He described that for patients under anesthesia in the prone position, the lungs expand better, and improved oxygenation could be observed [7]. Increased exhalation volume, better coordination of ventilation and blood circulation in the lungs, transformation of the effective area of lung ventilation and interference in the variations of rib cage volume are the effects of the prone position in patients [8].

In a meta-analysis by Sad et al., entitled “Analysis of the effects of prone position on oxygenation of ARDS patients”, ventilation in the prone position increased the ratio of the oxygen of arterial blood to the inhalation oxygen percentage from 23 to 34% during the first three days [9]. In another research done in the public Hospital of Marseille in 2016, entitled “Oxygenation in response to the prone position in patients with acute respiratory failure under mechanical ventilation”, by Haddam et al., the results of the study showed that the prone position had no effect on patients’ oxygenation [10].

Since the nursing personnel deals more with the patients and due to the importance of this caring unit, taking full care of the patients under MV is a significant factor in nursing. Therefore, in addition to utilization of MV, a supportive intervention plan must be designed to improve the oxygenation quality and other caring measures for the patients and to control the effect of treatment and prevention of the side effects of MV. Although many books recommend that patients are put in the prone position, worries about its probable side effects in terms of cardiac and respiratory problems have practically discouraged nurses from using that position in ICUs.

Since nowadays emphasis is put on evidence-based nursing, making effective use of nursing measures like the prone position, specifically in the ICU with its unstable and semi-stable hemodynamic patients, requires a lot of research regarding effects, effectiveness, and probable hazards for the patients. So, the present study was performed aiming at investigating the effects of the prone position on oxygenation of patients with ARF under MV.

## Methods

The present study is a single-group clinical trial (pretest-posttest), which was performed in 2017 in Ahvaz City. Sampling was made after approval of the project and acknowledgment by the Ethical Committee of the Ahvaz City University of Medical Sciences with the code IR.AJUMS.REC.139.96, and agreement of the authorities of Golestan Hospital. The population of the study included all patients with ARF under MV in ICU in the Golestan Hospital of Ahvaz City.

Inclusion criteria were as follows: Having ARF (Pao_2_<50, paco_2_>50), 18-60 years of age, having had tracheal intubation for at least 6 hours, being on the Synchronized Intermittent Mechanical Ventilation, being hemodynamically stable, lacking any symptoms of pressure ulcers, specifically mild edema (+1), any scratch or inflammation of skin which are primary symptoms of pressure ulcers in the shoulder, face, toes, and iliac area, and also increased intracranial pressure.

Exclusion criteria were: Undesirable variation of heart rate (less than 60 or higher than 100), undesirable variation of blood pressure (systolic pressure above 140 and diastolic pressure below 60 mmHg), decreased saturation of arterial blood oxygen (less than 80%), emergence of ventricular tachycardia, asystole and ventricular fibrillation, need for variation of ventilation mode.

Demographic, clinical and physiological data were collected and included age, gender, mechanical ventilation duration, the cause of hospitalization, and history of previous diseases, blood pressure, respiratory rate and pulse rate, arterial blood oxygen and the saturation percentage of arterial blood oxygen.

A content validation methodology was used to determine the validity of the demographic form, clinical form, and the physiological checklist. According to the objectives of the study, after preparation, the forms were reviewed by ten faculty members of the Nursing School of Ahvaz City University of Medical Sciences. The data gathering instrument was validated after applying their opinions and recommendations and after approval by the supervisor and the advisor. The validity of cardiac monitoring, mechanical ventilation, and ABG testing instruments was approved by referring to the manufacturer manual and calibration. In order to improve the reliability of the physiological indices, use was made of the Sadaat cardiac monitoring device model Alborz b9, Drager Savina and Inspiration mechanical ventilation instruments made by Event, and TECHNO MEDICA ABG testing device CASTAT 1800 Series for all patients.

Every patient was investigated for three days. On day one, the patient was placed in the supine position for two hours. The physiological indices (blood pressure, respiration, heart rate) of the patients were measured continuously for 2 hours.

At the end of the supine position stage, an arterial blood sample was taken from the patients for ABG tests and sent to the laboratory. For this stage, the radial, femoral or brachial artery was found by its pulse rate, and the sampling area was disinfected with Betadine (center-outward rotational movement was used).

In cases where the radial artery was used for sampling, Allen’s test was performed before sampling to analyze the blood circulation. In this study, the patients were not conscious. The artery was found by index and middle fingers, and the vessel was held between the two. A heparinized needle (heparin was drawn by the syringe, it was held upwards, the piston was pulled back to soak the entire syringe with heparin and then the entire heparin was removed slowly) was entered at an angle of 30-45° in blood sampling from radial and brachial arteries and at an angle of 60° in sampling from the femoral artery. The needle was entered once, and 2cc of the blood was drawn. After blood sampling, a gauze was put and held in place for 5 min. In case the patient used anti-coagulation drugs, the gauze time was extended to 10-15 minutes. Air bubbles were removed from the syringe and the needle was blocked with a plastic cap, so that air could not enter. The samples were labeled and transferred to the laboratory in ice bags. The vital signs of the patients were analyzed and any symptoms of dysfunction in blood circulation, such as inflammation, color change, numbness or hemorrhage at the blood sampling area were investigated [12].

After two hours, the patient was soon placed in a prone position. Before that, the oral secretions or tracheal tube contents were removed by suction. Also, tracheal tube cuff inflation was ensured to prevent its movement or ejection when changing positions. It was fastened with the band provided.

Three people were needed to place the patient in a prone position. One of them took care of the patient’s head, tracheal tube, and ventilation tubing. Two others were on either side. In the first step, the rotation direction (right/left) was determined and priority was given to the central venous tubes; afterward, the ventilation and vascular tubes were realigned, and the tracheal tube was held tight. Then, the patient was rotated (right or left) into the lateral position, and the position was controlled shortly until the cardiac electrodes were transferred to his/her back. Finally, the patient was in a prone position.

The physiological signs of the patient were measured at one-hour intervals during these two hours. The ABG sampling was made again by the nursing specialist according to the stated principles. The samples were sent to the laboratory. The patients, while in the prone position, were investigated in terms of specific cardiac variations.

One patient was excluded from the study due to an increase in the heart rate (124/min) 15 minutes after turning into the prone position. One patient started to remove the mechanical ventilation equipment 15 minutes after turning into the prone position. Another patient encountered increased respiratory rate (32/min) and was, again, excluded. Another patient was excluded due to decreased blood pressure (87.60). The required medical and nursing measures were taken, and the patients were excluded from the study.

## Results

According to Table 1, the paired t-test showed that there is a significant difference between the oxygen saturation percentage of the arterial blood and the prone position of the patient during the first three days. On day one, the prone position had no effects on the improvement of oxygen saturation of the arterial blood. Although there is a statically significant relationship between the average oxygen saturation of the arterial blood before and after the intervention (p<0.05), the relation is not aligned with the objectives of the study. On days two and three, the prone position had a positive effect on the improvement of oxygen saturation of the arterial blood; and there is a significant relationship between the average variations of the index before and after the intervention (p<0.05). So, on day one, the average oxygen saturation of the arterial blood was 93.74 in the supine position and 92.24 in the prone position, which is statically significant (p<0.05). However, this is not a significant difference in clinical terms, and on days 2 and 3, the average oxygen saturation of the arterial blood was 93.92 and 95.27, respectively. These values are lower than the values of the average oxygen saturation of the arterial blood in a prone position during days two and three (95.4 and 96.72, respectively).

**Table 1:** Comparison of average sao_2_ for the patients before and after intervention during three consecutive days

Time	Day one	Day two	Day three	p-value repeated measurement
**Sao_2_**	Mean ±SD	Mean ±SD	Mean ±SD	<0.05
**Supine**	95.46±7.33	95.69±7.48	97.73±7.74	
**Prone**	93.76±7.56	97.82±7.49	99.45±7.83	
**p-value pair T-test**	<0.05	<0.05	<0.05	

Also, according to Table 2, the partial pressure of oxygen in the arterial blood and the prone position of the patients during the three days had a significant relation. On day one there was a fall in pao_2_ followed by an increase and both were statistically significant. However, this relation is not aligned with the objectives of the study. On days 2 and 3, the prone position had a positive effect of the partial pressure of oxygen in the arterial blood, the differences of which were significant before and after the intervention (p<0.05). As observed, on day one, the average partial pressure of oxygen in the arterial blood was 95.46 in the supine and 93.76 in the prone positions which was statistically significant (p<0.05). However, these are not significant differences in clinical terms. The average partial pressure of oxygen in the arterial blood was 95.69 and 97.73 in the supine position on the days two and three, which were less than the average partial pressure of oxygen in the arterial blood in the prone position during the same days (97.82 and 99.46, respectively).

**Table 2:** Comparison of average pao_2_ for the patients before and after intervention during three consecutive days

Time	Day one	Day two	Day three	p-value repeated measurement
**Pao_2_**	Mean ±SD	Mean ±SD	Mean ±SD	<0.05
**Supine**	93.74±1.82	93.92±1.46	95.27±1.17	
**Prone**	92.24±2.008	95.40±1.23	96.72±1.12	
**p-value pair T-test**	<0.05	<0.05	<0.05	

According to Table 3, on day one, the average systolic blood pressure in the prone position was 0.56, higher compared to the supine position. By the paired t-test, this is not a significant difference (p-value>0.05).

**Table 3:** Comparison of average Systolic Blood Pressure for the patients before and after intervention during three consecutive days

Time	Day one	Day two	Day three	p-value repeated measurement
**Systolic Blood Pressure**	Mean ±SD	Mean ±SD	Mean ±SD	0/842
**Supine**	122/77±12/19	121/57±8/09	120/86±9/23	
**Prone**	123/33±7/4	121/32±8/71	123/5±7/89	
**p-value pair T-test**	0/644	0/688	0/02	

According to Table 4, by the paired t-test, there is a significant difference (p-value<0.05) between average Diastolic Blood Pressure for the patients before and after intervention during three consecutive days.

**Table 4:** Comparison of average Diastolic Blood Pressure for the patients before and after intervention during three consecutive days.

Time	Day one	Day two	Day three	p-value repeated measurement
**Diastolic Blood Pressure**	Mean ±SD	Mean ±SD	Mean ±SD	0/768
**Supine**	6/97±0/33	7/44±0/37	7/2±0/36	
**Prone**	6/55±0/39	6/77±0/44	6/56±0/48	
**p-value pair T-test**	<0/05	<0/05	<0/05	

As seen in Table 5, the average respiratory rate in the prone position was 0.42 higher compared to the supine position, which was, again, not significant with respect to the paired t-test (p-value>0.05). Moreover, the average heart rate was 0.89 lower in the prone position than supine position, which was significant, based on the paired t-test (p-value<0.05). On day two, the average blood pressure was 0.25 less in the prone position compared to the supine position. This is not a significant difference based on the paired t-test (p-value>0.05). The average respiratory rate in the prone position was 0.35 higher than the supine position which was not significant according to the paired t-test (p-value>0.05).

**Table 5:** Comparison of average Respiratory rate for patients before and after intervention during three consecutive days.

Time	Day one	Day two	Day three	p-value repeated measurement
**Respiratory rate**	Mean ±SD	Mean ±SD	Mean ±SD	0/655
**Supine**	18/54±1/14	18/53±1/31	18/38±1/28	
**Prone**	18/97±2/005	18/89±1/81	19/12±1/95	
**p-value pair T-test**	>0/05	>0/05	<0/05	

According to Table 6, the average pulse rate in the prone position was 0.75 less than in the supine position. This difference was not significant according to the paired t-test (p-value>0.05). On the third day, the average blood pressure was 2.64 higher in the prone position than in the supine position. This is a significant difference based on the paired t-test. The average respiratory rate in the prone position was 0.74 higher compared to the supine position which was significant, based on the paired t-test. Finally, the average heart rate in the prone position was 1.39 lower compared to the supine position. This difference was significant according to the paired t-test. The results show that on day three, all variables had significant differences as opposed to day one when only the heart rate variable had a significant difference (p-value<0.05).

**Table 6:** Comparison of average pulse rate for patients before and after intervention during three consecutive days.

Time	Day one	Day two	Day three	p-value repeated measurement
**Pulse rate**	Mean ±SD	Mean ±SD	Mean ±SD	0/556
**Supine**	77/66±9/17	78/81±10/15	78/66±9/31	
**Prone**	76/76±9/8	78/06±10/007	77/27±9/11	
**p-value pair T-test**	<0/05	>0/05	<0/05	

## Discussion

A statistically significant difference in saturation was noted from day one. On the first day, this was lower and on the other two days higher in prone than in the supine position. In this regard, Bassam Poor et al. investigated the effect of the prone position on oxygenation and the physiological signs in 36 patients with acute respiratory failure under mechanical ventilation. The results showed no significant relationship between the prone position and physiological signs[11].

According to the results obtained by our study, the prone position may improve the oxygen pressure of the arterial blood and the oxygen saturation of the arterial blood. In the studies mentioned hereafter, the effect of the duration of being placed in prone position on pao_2_ and sao_2_ are investigated.

In a study by Black and Hawks, entitled “The effects of prone position in patients with hydrostatic edema and patients with acute respiratory distress syndrome and pulmonary fibrosis”, a significant increase of sao_2_ was observed in 75% of patients with respiratory failure after 30 minutes in the prone position [13].

Keizer et al., in a study entitled “Analysis of short-term effects of nitric acid and prone position on patients with acute respiratory distress syndrome”, stated that 30 minutes after getting in prone position, 80% of patients had a 20mmHg increase in pao_2_; there was a statistically significant difference between this and the basic value [14].

In a study in Al-Zahra Hospital of Isfahan Province, entitled “Effects of the prone position on cardiac and respiratory indices in patients under mechanical ventilation”, Yazdannik et al. observed a significant difference in spo2 under mechanical ventilation 30 minutes after getting the patients in the prone position. Although Yazdannik et al. used a Pulse Oximetry apparatus to measure the oxygen saturation percentage; any increase in the oxygen saturation percentage of the blood may indicate an increase in the oxygen amount dissolved in the blood [15].

In this regard, in the meta-analysis by Sad et al., entitled “Effects of prone position on oxygenation of patients with ARDS”, ventilation in the prone position increased the ratio of the oxygen of arterial blood to the inhalation oxygen percentage from 23 to 34% during the first three days [9].

In another study in the public Hospital of Marseille in 2016, entitled “Oxygenation in response to prone position in patients with acute respiratory failure under mechanical ventilation”, Haddam et al. selected 51 patients. In this study, the patients were put into the prone position for one-hour and into the supine position for another hour. The oxygenation of the patients was investigated in four stages (one hour before and one hour after getting into the prone position, and the same for the supine position). The results of the study showed that the prone position had no effects on patients’ oxygenation [10].

Bassampour et al. investigated the effect of the prone position on oxygenation in 36 patients with acute respiratory failure under mechanical ventilation hospitalized in Shariati Hospital of Tehran University of Medical Sciences who qualified for the study. The results showed that there is a significant relationship between the prone position and the saturation level of arterial blood for 30- and 120-minutes intervals; i.e., application of the prone position has a positive effect on oxygenation [11].

Abroug et al. published a meta-analysis that index six RCTs with data from 1372 patients. Ventilation in the prone position in this meta-analysis showed a significant improvement in pao_2_/fio_2_. The results of this meta-analysis do not justify the routine use of the prone position during MV in a patient with acute hypoxemic respiratory insufficiency, including acute lung injury and ARDS [16].

The RCT by Taccone et al. included 342 patients and showed a significantly higher frequency of adverse events in patients rested in the prone position [17].

Three studies with 827 participants reported improvement in oxygenation over seven to 10 days. The mean difference in improvement in the pao_2_ quotient was 24.6 mmHg compared with baseline measurement at study entry [18–20].

## Limitations

Every study has its limitations which emerge during the realization of the study and creates challenges. The present study was not an exception. Here, limitations included a uniform study group of patients (due to the presence of a single group) and insufficient facilities such as the beds required for putting the patients in the prone position.

## Conclusions and Recommendations

The present study indicates that the prone position improves the oxygen pressure of the arterial blood and the oxygen saturation level of the arterial blood in patients with acute respiratory failure under mechanical ventilation. In other words, in order to improve the oxygenation of the arterial blood in patients with acute respiratory failure under mechanical ventilation, we are able to not only increase the delivered partial oxygen and vary the respiratory mode, ratio of inhalation/exhalation, etc., but also to comply with the indices introduced in the “inclusion indices” and to apply the prone position for two hours per day. Considering the results of the present study, the nursing personnel of the ICU can be recommended to use this position as a possible position for the patients, not only to prevent immobility side effects, but also to have positive effects on oxygenation. Also, the effect of this lying position on the time duration of separation of the patient from the mechanical ventilation apparatus, or its effect on the patients with chronic respiratory failure under mechanical ventilation could be investigated in the future studies.

## Acknowledgment

This study is part of the results of the thesis for M.S in Nursing School of Ahvaz City University of Medical Sciences with the code of IR.AJUMS.REC.139.96 and was sponsored by Ahvaz City University of Medical Sciences. The authors, hereby, acknowledge the above authorities and the families of participating patients and the ICU staff of Golestan Hospital of Ahvaz County.

## Conflict of Interest

The authors confirm that there are no conflicts of interest.
