# Correction to: Determination of the effects of prone position on oxygenation in patients with acute respiratory failure under mechanical ventilation in ICU

**DOI:** 10.25122/jml-2021-1008

**Published:** 2021

**Authors:** Simin Jahani, Ziba Hajivand Soleimani, Marziyeh Asadizaker, Farhad Soltani, Bahman Cheraghian

**Affiliations:** 1. Nursing and Midwifery School, Ahvaz Jundishapur University of Medical Sciences, Ahvaz, Iran; 2. Department of Anesthesia and Intensive Care Unit, School of Medicine, Ahvaz Jundishapur University of Medical Sciences, Ahvaz, Iran; 3. Health School, Ahvaz Jundishapur University of Medical Sciences, Ahvaz, Iran

## Abstract

This corrects the article on p. 274 in vol. 11, PMID: 30894882.

## Correction

In the original publication [1], the name and title of the coresponding author have been mistyped. The original article is now corrected. We wish to apologise for any misunderstanding or inconvenience caused.

## References

[1] Jahani S, Hajivand Solymani Z, Asadizaker M, Soltani F, Cheraghian B (2018). Determination of the Effects of Prone Position on Oxygenation in Patients with Acute Respiratory Failure Under Mechanical Ventilation in ICU.. J Med Life.

